# Notes and News

**Published:** 1894-04-14

**Authors:** 


### Notes and News.

Collected and Communicated.

MEETINGS OF THE WEEK.

German Hospital, Dalston, Festival Dinner, Hôtel Metropole, Thursday, April 12th.
Middlesex Hospital, Festival Dinner, Hôtel Metropole, Friday, April 13th.
Seamen's Hospital, Greenwich, Quarterly General Court, 29, Budge Row, Friday, April 13th, 3·0 p.m.

The National Lying-in Hospital at Dublin has been reopened. An appeal has appeared in the Dublin papers for funds, which has met with good response. The hospital is essentially Roman Catholic in character, and this point will not be lost sight of in collecting subscriptions. The charity is an undoubtedly useful one, and serves a district untouched by the Rotunda and Coombe hospitals.

The Duke of Cambridge has fixed Thursday, May 3rd, as the date at which he will preside at a public meeting to be held at Prince's Hall for promoting a memorial to the late Sir A. Clark, and Mr. Gladstone will speak at the meeting. It is intended that the memorial shall take the form of an Andrew Clark Wing of the London Hospital, in which some necessary extensions of certain departments of the hospital will be made.

The International Sanitary Convention, held at Paris, has finished its deliberations. As was understood, the chief subject under consideration was the regulations respecting the Mecca Pilgrims and the spread of infection through them. The Convention has effected that the voyagers shall be closely watched throughout their journeys, and all necessary precautions taken. At the same time Turkey is to discontinue putting healthy vessels into quarantine, and admitting for the future only a short detention of 48 hours, in order to examine the pilgrims and disinfect their personal effects. A new body is to be formed to carry out the terms of the Convention within the Council of Constantinople. The whole question of the pilgrimage is now on a better footing. England has consented to a number of conditions, and in return has gained many concessions for the pilgrims, and a body has been secured to deal with these matters imbued with modern sanitary requirements.

A cottage hospital has just been opened at Ashby-de-la-Zouch. It is intended for the use of the town and district, and was opened by Lady Cave. The building, now ready to accommodate six adults and three children, is held on lease from Lord Donington, but it is hoped ultimately to erect a permanent hospital.

A hospital has just been opened at Udaipur, India. It is called the Lansdowne Hospital, in commemoration of Lord Lansdowne's visit, when Viceroy, to the Rajput States. It was built by the Maharana, and was declared open by the Governor-General's Agent in Rajputana. The architecture is essentially Indian. It is built of stone, the facade being flanked on each side by high towers, in the building of which some beautiful tracery has been used. The ground floor is occupied by receiving-room, dispensary, operating-room, and wards; the upper storey contains pay and other wards.

The new Derbyshire Infirmary is progressing towards completion. Most of the work promises to be ready in three months. The administration block is the least forward, whilst the dispensary is practically finished. At the last meeting of the infirmary an expenditure of £5,000 was sanctioned, to provide new roads, the laying out of the grounds, and the removal of some of the old drains. At the same meeting one of the governors pleaded very hard for the general use of electric light throughout the buildings. The suggestion was vetoed on the ground of expense, but all arrangements are to be made for its application should funds be forthcoming or rates diminish.

At the annual meeting of the Birmingham Hospital Saturday Fund, the speakers, after dwelling in detail on the statistics of the Fund, spoke with enthusiasm of the generosity of the working classes towards the medical charities. The collection of last year enabled the committee to hand over £10,000 to the hospitals, and reserve over £1,100 for the Men's Convalescent Home at Tyn-y-Coed. Alderman Cook dwelt with satisfaction on the management of the Fund, taking the London Fund as a subject of comparison. The expenses of the Birmingham Fund, he pointed out, amounted to 4·37 per cent. of the collection, whilst that of London amounted to 13·5 per cent. Again he showed that the greater economy was in respect to the respective convalescent homes of the two cities was shown by that of Birmingham. At this meeting it was decided to establish the proposed Convalescent Home for Women at Marle Hall, near Llandudno.

The new General Hospital at Birmingham, of which the Duke of York has promised to lay the foundation-stone this year, is estimated to cost £200,000, a larger sum than was at first deemed necessary. The site was purchased for £50,000, and £6,000 is yet unsubscribed to complete the undertaking. By the Wilks bequest the General Hospital became the possessor of £45,000. The bye-laws of the institution require that half the amount accruing from legacies shall be placed to the capital of the hospital. In the case of the Wilkes bequest this leaves £22,000 unhampered by restrictions. The governors of the hospital, with the approval of the Finance Committee, have decided to devote this sum to the Building Fund, hoping that the public will then come forward liberally to subscribe the rest. The Ryland bequest of £25,000 will go some way towards supplying the increased income which will doubtless be needed when the new and enlarged hospital is completed.

